# {μ-Bis(1-methyl­imidazol-2-yl)methane-κ^2^ *N* ^3^:*N* ^3′^}bis­{[(1-methyl­imidazol-2-yl)methane-κ^2^ *N* ^3^,*N* ^3′^]copper(I)} bis­(tri­fluoro­methane­sulfonate)

**DOI:** 10.1107/S1600536809039622

**Published:** 2009-10-03

**Authors:** Jun Matsumoto, Yuji Kajita, Hideki Masuda

**Affiliations:** a Department of Applied Chemistry, Nagoya Institute of Technology, Showa-ku, Nagoya 466-8555, Japan

## Abstract

The title compound, [Cu_2_(C_9_H_12_N_4_)_3_](CF_3_SO_3_)_2_, contains two Cu^I^ ions, three bis­(1-methyl­imidazol-2-yl)methane (Me_2_BIM) ligands, and two trifluoromethanesulfonate anions in the asymmetric unit. Each Cu^I^ ion has a distorted trigonal-planar geometry  and is coordinated by two N atoms from the Me_2_BIM ligand and another N atom from the Me_2_BIM that acts as a bridging ligand, another N atom of the bridging Me_2_BIM being linked to the second Cu^I^ ion. The imidazole rings of Me_2_BIM form intra­molecular π–π stacking inter­actions [centroid–centroid distances = 3.445 (2) and 3.547 (2) Å].

## Related literature

For the protonated ligand Me_2_BIM, see: Messerle *et al.* (2003 ▶). For coordination complexes with one or two Me_2_BIM ligands chelating one metal center, see: Elgafi *et al.* (1999 ▶); Abuskhuna *et al.* (2004*a* ▶); Burling *et al.* (2004 ▶); Kennedy *et al.* (2007 ▶); Dabb *et al.* (2009 ▶). For Cu^II^ complexes with two BIM ligands chelating one metal center, see: Place *et al.* (1998 ▶). For Ag^I^ complexes with two BIM ligands bridging two metals, see: Abuskhuna *et al.* (2004*b* ▶). The Me_2_BIM ligand was synthesized by modified literature methods (Byers & Canty, 1990 ▶; Elgafi *et al.*, 1997 ▶).
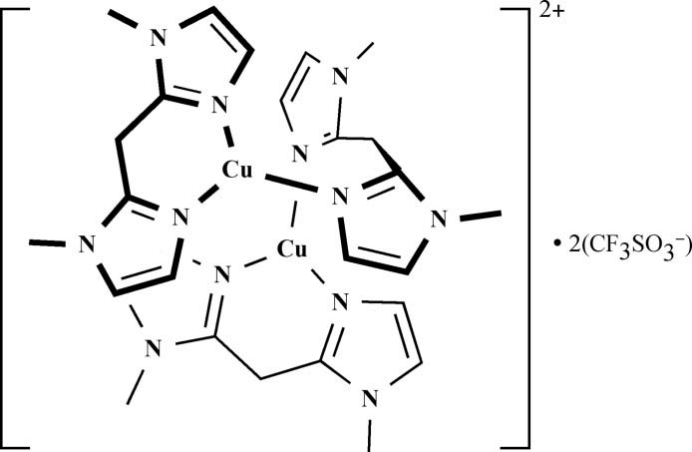


## Experimental

### 

#### Crystal data

[Cu_2_(C_9_H_12_N_4_)_3_](CF_3_SO_3_)_2_
*M* *_r_* = 953.88
Monoclinic, 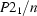

*a* = 22.5575 (11) Å
*b* = 7.0307 (3) Å
*c* = 25.4982 (16) Å
β = 109.118 (2)°
*V* = 3820.9 (3) Å^3^
*Z* = 4
Mo *K*α radiation
μ = 1.31 mm^−1^
*T* = 173 K
0.20 × 0.15 × 0.15 mm

#### Data collection

Rigaku Mercury diffractometer
Absorption correction: none
29376 measured reflections
8745 independent reflections
6639 reflections with *I* ^2^ > 2σ(*I* ^2^)
*R*_int_ = 0.039

#### Refinement

*R*[*F* ^2^ > 2σ(*F* ^2^)] = 0.055
*wR*(*F* ^2^) = 0.146
*S* = 1.14
8745 reflections
515 parameters
H-atom parameters constrained
Δρ_max_ = 0.58 e Å^−3^
Δρ_min_ = −0.72 e Å^−3^

### 

Data collection: *CrystalClear* (Rigaku, 2007 ▶); cell refinement: *CrystalClear*; data reduction: *CrystalStructure* (Rigaku, 2007 ▶); program(s) used to solve structure: *SIR2004* (Burla *et al.*, 2005 ▶); program(s) used to refine structure: *SHELXL97* (Sheldrick, 2008 ▶); molecular graphics: *ORTEP-3 for Windows* (Farrugia, 1997 ▶); software used to prepare material for publication: *CrystalStructure*.

## Supplementary Material

Crystal structure: contains datablocks global, I. DOI: 10.1107/S1600536809039622/bv2129sup1.cif

Structure factors: contains datablocks I. DOI: 10.1107/S1600536809039622/bv2129Isup2.hkl

Additional supplementary materials:  crystallographic information; 3D view; checkCIF report

## Figures and Tables

**Table:** 

Cu1—N1	2.042 (2)
Cu1—N3	1.958 (3)
Cu1—N9	1.914 (2)
Cu2—N5	2.049 (3)
Cu2—N7	1.935 (3)
Cu2—N11	1.916 (3)

**Table:** 

N1—Cu1—N3	95.56 (12)
N1—Cu1—N9	121.37 (12)
N3—Cu1—N9	142.95 (12)
N5—Cu2—N7	93.66 (12)
N5—Cu2—N11	115.27 (11)
N7—Cu2—N11	150.78 (12)

**Fig. 1.**
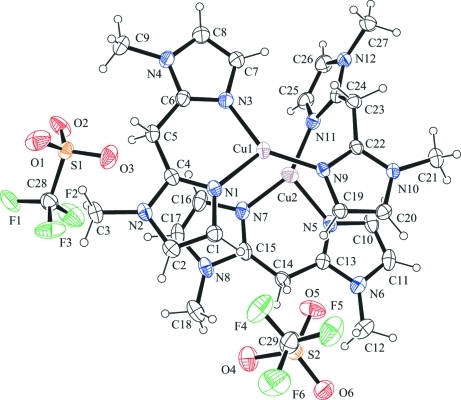
The molecular view of the title compound with the atomic numbering scheme. Displacement ellipsoids are drawn at the 30% probability level.

**Fig. 2.**
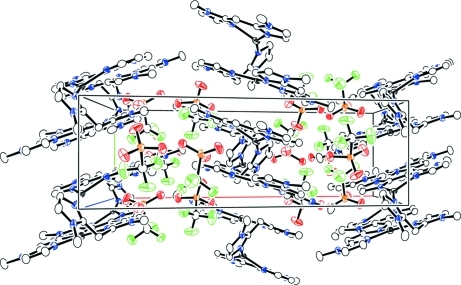
The crystal packing of the title compound viewed along the c axis. Displacement ellipsoids are drawn at the 30% probability level. Hydrogen atoms that are not involved in hydrogen bonding are omitted for clarity.

**Fig. 3.**
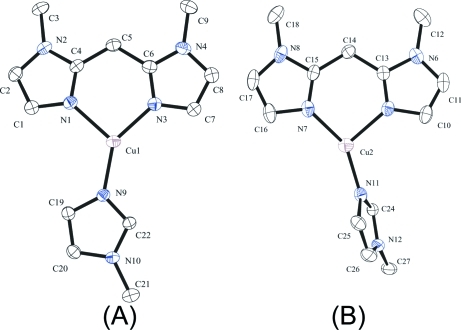
The structures of (A) and (B) show the circumferences of the coppers center, Cu(1) and Cu(2), respectively. Displacement ellipsoids are drawn at the 30% probability level. Hydrogen atoms that are not involved in hydrogen bonding have been omitted for clarity.

## supplementary crystallographic information

### Comment

Ligand bis(imidazol-2-yl)methane derivative is a bidentate ligand, either chelating one metal center or bridging two metals. The crystal structures of Cu^II^ complexes with bis(imidazol-2-yl)methane (BIM) and bis(1-methylimidazol-2-yl)methane (Me_2_BIM) have been reported by Place *et al.* (1998), Abuskhuna *et al.* (2004*a*), respectively. In addition, that of Ag^I^ complex with BIM has been reported by Abuskhuna *et al.* (2004*b*). The Cu^II^ complexes have four- or six-coordinate structure that is coordinated with two Me_2_BIM ligands, and the Ag^I^ complex forms a two-coordinate structure bridged by BIM. The title compound, [(µ-Me_2_BIM){Cu(Me_2_BIM)}_2_](OTf)_2_, contains two Cu^I^ ions, three bis(1-methylimidazol-2-yl)methane (Me_2_BIM) ligands, and two trifluoromethanesulfonate anions as an independent unit in the unit cell. Each Cu^I^ ion has a distorted trigonal planar geometry coordinated with two N atoms from Me_2_BIM and one N atom from a bridging Me_2_BIM ligand, and an N atom from an additional bridging Me_2_BIM which is linked to another Cu^I^ ion. This is a novel dinuclear Cu^I^ complex with Me_2_BIM. An intramolecular π—π stackings are formed between the imidazol rings; angles between the imidazole ring 1 containing N(1) and the ring 2 containing N(7) and between the ring 3 containing N(5) and the ring 4 containing N(9) are 4.4 (1) and 2.2 (2)°, respectively, and distances of the centroid of plane 1 to plane 2 and that of plane 3 to plane 4 are 3.445 (2) and 3.547 (2) Å, respectively.

### Experimental

Ligand Me_2_BIM was synthesized by modifying the literature methods of Byers *et al.* (1990) and Elgafi *et al.* (1997). Ligand Me_2_BIM (1.13 mmol, 200 mg) in CH_2_Cl_2_ (4 ml) was added into a MeCN solution (3 ml) of [Cu(MeCN)_4_](OTf) (1.15 mmol, 433 mg). The mixture was stirred for 1 h at room temperature in a glovebox ([O_2_] < 1 p.p.m. and [H_2_O] < 1 p.p.m.). After addition of Et_2_O (7 ml), the resulting precipitates were collected by filtration and dried to give an yellow powder in a 65% yield. Single crystals suitable for X-ray crystallographic analysis were obtained by recrystallization from CH_2_Cl_2_/MeCN/Et_2_O.

### Refinement

Hydrogen atoms attached to carbon atoms were positioned geometrically and treated as riding with aromatic C—H = 0.95 Å, methyl C—H = 0.98 Å, and methylenic C—H = 0.99 Å and with *U*_iso_(H) = 1.2*U*_eq_(C).

### Crystal data

**Table:**

[Cu_2_(C_9_H_12_N_4_)_3_](CF_3_SO_3_)_2_	*F*(000) = 1944.00
*M**_r_* = 953.88	*D*_x_ = 1.658 Mg m^−^^3^
Monoclinic, *P*2_1_/*n*	Mo *K*α radiation, λ = 0.71070 Å
Hall symbol: -P 2yn	Cell parameters from 10516 reflections
*a* = 22.5575 (11) Å	θ = 3.0–27.5°
*b* = 7.0307 (3) Å	µ = 1.31 mm^−^^1^
*c* = 25.4982 (16) Å	*T* = 173 K
β = 109.118 (2)°	Block, yellow
*V* = 3820.9 (3) Å^3^	0.20 × 0.15 × 0.15 mm
*Z* = 4	

### Data collection

**Table:**

Rigaku Mercury diffractometer	*R*_int_ = 0.039
Detector resolution: 7.31 pixels mm^-1^	θ_max_ = 27.5°
ω scans	*h* = −29→23
29376 measured reflections	*k* = −9→9
8745 independent reflections	*l* = −27→33
6639 reflections with *F*^2^ > 2σ(*F*^2^)	

### Refinement

**Table:**

Refinement on *F*^2^	0 restraints
*R*[*F*^2^ > 2σ(*F*^2^)] = 0.055	All H-atom parameters refined
*wR*(*F*^2^) = 0.146	*w* = 1/[σ^2^(*F*_o_^2^) + (0.0583*P*)^2^ + 1.5952*P*] where *P* = (*F*_o_^2^ + 2*F*_c_^2^)/3
*S* = 1.14	(Δ/σ)_max_ = 0.001
8745 reflections	Δρ_max_ = 0.58 e Å^−^^3^
515 parameters	Δρ_min_ = −0.72 e Å^−^^3^

### Special details

**Table:**

Refinement. Refinement was performed using all reflections. The weighted *R*-factor (*wR*) and goodness of fit (*S*) are based on *F*^2^. *R*-factor (gt) are based on *F*. The threshold expression of *F*^2^ > 2.0 σ(*F*^2^) is used only for calculating *R*-factor (gt).

### Fractional atomic coordinates and isotropic or equivalent isotropic displacement parameters (Å^2^)

**Table:**

	*x*	*y*	*z*	*U*_iso_*/*U*_eq_	
Cu(1)	0.525378 (19)	0.21542 (6)	0.763364 (17)	0.03927 (13)	
Cu(2)	0.52080 (2)	0.61525 (6)	0.716419 (19)	0.04633 (14)	
S(1)	0.81822 (4)	0.46106 (15)	0.93194 (4)	0.0487 (2)	
S(2)	0.34890 (4)	0.48802 (15)	0.89323 (4)	0.0475 (2)	
F(1)	0.87401 (14)	0.6587 (6)	1.02074 (11)	0.1071 (12)	
F(2)	0.8022 (2)	0.8084 (4)	0.96078 (14)	0.1107 (12)	
F(3)	0.77953 (15)	0.5968 (4)	1.01045 (15)	0.1008 (11)	
F(4)	0.38615 (13)	0.1387 (4)	0.91831 (15)	0.0937 (9)	
F(5)	0.28917 (13)	0.1643 (4)	0.87220 (15)	0.0935 (10)	
F(6)	0.32319 (18)	0.2407 (5)	0.95855 (15)	0.1125 (12)	
O(1)	0.83456 (19)	0.2915 (5)	0.96391 (16)	0.0920 (12)	
O(2)	0.86351 (15)	0.5278 (5)	0.90881 (12)	0.0753 (9)	
O(3)	0.75529 (14)	0.4666 (5)	0.89437 (15)	0.0845 (10)	
O(4)	0.40376 (14)	0.5429 (5)	0.93745 (14)	0.0859 (11)	
O(5)	0.35806 (17)	0.4652 (4)	0.84099 (12)	0.0761 (9)	
O(6)	0.29232 (13)	0.5838 (4)	0.89142 (13)	0.0627 (7)	
N(1)	0.54920 (13)	0.1991 (3)	0.84769 (11)	0.0379 (6)	
N(2)	0.61408 (14)	0.2013 (4)	0.93364 (11)	0.0406 (6)	
N(3)	0.60648 (13)	0.1266 (4)	0.76032 (12)	0.0416 (6)	
N(4)	0.70290 (13)	0.0161 (4)	0.78893 (13)	0.0461 (7)	
N(5)	0.44244 (14)	0.7760 (4)	0.70788 (12)	0.0413 (6)	
N(6)	0.36224 (14)	0.8792 (4)	0.73090 (14)	0.0474 (7)	
N(7)	0.55491 (14)	0.6557 (4)	0.79575 (13)	0.0449 (7)	
N(8)	0.55692 (15)	0.7111 (4)	0.88142 (13)	0.0489 (7)	
N(9)	0.44262 (12)	0.2866 (3)	0.71791 (11)	0.0342 (5)	
N(10)	0.36179 (12)	0.3562 (3)	0.64410 (11)	0.0359 (6)	
N(11)	0.52866 (13)	0.5210 (4)	0.64851 (12)	0.0409 (6)	
N(12)	0.52401 (13)	0.3560 (4)	0.57365 (12)	0.0449 (7)	
C(1)	0.51766 (17)	0.2603 (4)	0.88285 (15)	0.0418 (7)	
C(2)	0.55684 (17)	0.2619 (5)	0.93589 (15)	0.0441 (8)	
C(3)	0.67175 (18)	0.1869 (6)	0.98115 (15)	0.0542 (10)	
C(4)	0.60759 (15)	0.1658 (4)	0.87997 (13)	0.0362 (7)	
C(5)	0.66238 (16)	0.1017 (5)	0.86426 (14)	0.0410 (7)	
C(6)	0.65498 (16)	0.0818 (4)	0.80423 (15)	0.0395 (7)	
C(7)	0.62501 (18)	0.0857 (5)	0.71475 (15)	0.0500 (9)	
C(8)	0.68394 (18)	0.0176 (6)	0.73197 (16)	0.0536 (9)	
C(9)	0.76404 (18)	−0.0461 (7)	0.82589 (18)	0.0656 (12)	
C(10)	0.39374 (18)	0.8252 (5)	0.66014 (17)	0.0507 (9)	
C(11)	0.34477 (18)	0.8879 (5)	0.67389 (17)	0.0528 (10)	
C(12)	0.3231 (2)	0.9282 (6)	0.7644 (2)	0.0670 (12)	
C(13)	0.42209 (15)	0.8108 (4)	0.74969 (14)	0.0373 (7)	
C(14)	0.45525 (16)	0.7850 (5)	0.81032 (14)	0.0429 (8)	
C(15)	0.52150 (16)	0.7159 (4)	0.82688 (14)	0.0401 (7)	
C(16)	0.61394 (17)	0.6113 (5)	0.83269 (18)	0.0539 (10)	
C(17)	0.61495 (19)	0.6439 (5)	0.88480 (18)	0.0565 (10)	
C(18)	0.5355 (2)	0.7561 (6)	0.92820 (17)	0.0621 (11)	
C(19)	0.39341 (15)	0.3483 (4)	0.73479 (14)	0.0373 (7)	
C(20)	0.34333 (16)	0.3899 (4)	0.68955 (15)	0.0404 (7)	
C(21)	0.32136 (18)	0.3793 (5)	0.58651 (15)	0.0524 (9)	
C(22)	0.42150 (15)	0.2945 (4)	0.66262 (13)	0.0339 (6)	
C(23)	0.45787 (16)	0.2303 (5)	0.62668 (15)	0.0414 (7)	
C(24)	0.50238 (15)	0.3732 (5)	0.61708 (14)	0.0377 (7)	
C(25)	0.56876 (17)	0.6003 (5)	0.62356 (17)	0.0502 (9)	
C(26)	0.56674 (18)	0.4982 (6)	0.57821 (18)	0.0586 (10)	
C(27)	0.50716 (19)	0.2054 (6)	0.53201 (17)	0.0612 (11)	
C(28)	0.8175 (2)	0.6401 (6)	0.98281 (17)	0.0580 (10)	
C(29)	0.3366 (2)	0.2461 (7)	0.9112 (2)	0.0617 (11)	
H(3A)	0.6629	0.2203	1.0152	0.065*	
H(3B)	0.7032	0.2745	0.9761	0.065*	
H(3C)	0.6877	0.0564	0.9841	0.065*	
H(9A)	0.7932	−0.0578	0.8048	0.079*	
H(9B)	0.7597	−0.1698	0.8420	0.079*	
H(9C)	0.7803	0.0474	0.8557	0.079*	
H(12A)	0.2814	0.9670	0.7400	0.080*	
H(12B)	0.3424	1.0333	0.7894	0.080*	
H(12C)	0.3190	0.8174	0.7863	0.080*	
H(18A)	0.5687	0.7257	0.9630	0.074*	
H(18B)	0.4980	0.6811	0.9254	0.074*	
H(18C)	0.5254	0.8919	0.9275	0.074*	
H(21A)	0.3013	0.5046	0.5818	0.063*	
H(21B)	0.2891	0.2800	0.5772	0.063*	
H(21C)	0.3466	0.3688	0.5619	0.063*	
H(27A)	0.5262	0.2318	0.5033	0.073*	
H(27B)	0.4614	0.2002	0.5151	0.073*	
H(27C)	0.5226	0.0832	0.5497	0.073*	
H(5A)	0.6759	−0.0232	0.8821	0.049*	
H(5B)	0.6971	0.1923	0.8807	0.049*	
H(14A)	0.4308	0.6940	0.8247	0.051*	
H(14B)	0.4550	0.9084	0.8289	0.051*	
H(23A)	0.4279	0.1925	0.5902	0.050*	
H(23B)	0.4820	0.1155	0.6436	0.050*	
H(1)	0.4747	0.2960	0.8714	0.050*	
H(2)	0.5470	0.2973	0.9680	0.053*	
H(7)	0.5998	0.1031	0.6771	0.060*	
H(8)	0.7077	−0.0216	0.7093	0.064*	
H(10)	0.3950	0.8157	0.6234	0.061*	
H(11)	0.3056	0.9303	0.6491	0.063*	
H(16)	0.6482	0.5650	0.8226	0.065*	
H(17)	0.6494	0.6240	0.9178	0.068*	
H(19)	0.3946	0.3597	0.7722	0.045*	
H(20)	0.3034	0.4337	0.6893	0.049*	
H(25)	0.5939	0.7100	0.6364	0.060*	
H(26)	0.5904	0.5207	0.5541	0.070*	

### Atomic displacement parameters (Å^2^)

**Table:**

	*U*^11^	*U*^22^	*U*^33^	*U*^12^	*U*^13^	*U*^23^
Cu(1)	0.0346 (2)	0.0410 (2)	0.0372 (2)	−0.00196 (17)	0.00483 (17)	0.00048 (18)
Cu(2)	0.0499 (2)	0.0403 (2)	0.0498 (2)	−0.00973 (19)	0.0176 (2)	−0.0088 (2)
S(1)	0.0388 (4)	0.0551 (5)	0.0516 (5)	0.0004 (4)	0.0142 (4)	0.0076 (4)
S(2)	0.0425 (5)	0.0587 (5)	0.0429 (5)	−0.0080 (4)	0.0163 (4)	−0.0085 (4)
F(1)	0.0700 (18)	0.202 (3)	0.0457 (15)	−0.017 (2)	0.0142 (13)	−0.0382 (19)
F(2)	0.188 (3)	0.0547 (17)	0.085 (2)	0.000 (2)	0.039 (2)	−0.0057 (16)
F(3)	0.103 (2)	0.100 (2)	0.140 (2)	−0.0088 (18)	0.095 (2)	−0.017 (2)
F(4)	0.0673 (18)	0.079 (2)	0.135 (2)	0.0213 (15)	0.0337 (18)	0.0262 (19)
F(5)	0.0582 (16)	0.0613 (16)	0.148 (2)	−0.0143 (13)	0.0164 (17)	−0.0128 (18)
F(6)	0.129 (2)	0.132 (3)	0.104 (2)	0.016 (2)	0.076 (2)	0.050 (2)
O(1)	0.127 (3)	0.072 (2)	0.095 (2)	0.047 (2)	0.061 (2)	0.038 (2)
O(2)	0.077 (2)	0.111 (2)	0.0459 (16)	−0.0351 (19)	0.0308 (14)	−0.0173 (17)
O(3)	0.0433 (16)	0.079 (2)	0.109 (2)	−0.0067 (15)	−0.0063 (16)	−0.019 (2)
O(4)	0.0546 (19)	0.105 (2)	0.084 (2)	−0.0264 (18)	0.0035 (16)	−0.033 (2)
O(5)	0.107 (2)	0.084 (2)	0.0552 (17)	0.0041 (19)	0.0506 (18)	0.0037 (16)
O(6)	0.0498 (16)	0.0592 (17)	0.078 (2)	−0.0022 (13)	0.0193 (14)	−0.0176 (15)
N(1)	0.0391 (15)	0.0367 (14)	0.0355 (14)	−0.0044 (11)	0.0091 (12)	−0.0003 (12)
N(2)	0.0475 (16)	0.0373 (15)	0.0316 (14)	−0.0037 (12)	0.0055 (12)	0.0020 (12)
N(3)	0.0387 (15)	0.0454 (16)	0.0394 (15)	0.0008 (12)	0.0109 (12)	0.0008 (13)
N(4)	0.0367 (15)	0.0469 (17)	0.0494 (17)	0.0066 (13)	0.0071 (13)	0.0001 (14)
N(5)	0.0436 (16)	0.0340 (14)	0.0393 (16)	−0.0042 (12)	0.0039 (12)	−0.0025 (12)
N(6)	0.0399 (16)	0.0362 (15)	0.063 (2)	−0.0016 (12)	0.0133 (14)	0.0012 (14)
N(7)	0.0379 (15)	0.0330 (14)	0.0543 (18)	−0.0078 (12)	0.0023 (13)	−0.0037 (13)
N(8)	0.0566 (19)	0.0388 (16)	0.0391 (16)	−0.0131 (14)	−0.0010 (14)	0.0003 (13)
N(9)	0.0340 (13)	0.0348 (14)	0.0314 (13)	−0.0053 (11)	0.0075 (11)	−0.0033 (11)
N(10)	0.0323 (13)	0.0367 (14)	0.0350 (14)	−0.0013 (11)	0.0057 (11)	−0.0002 (11)
N(11)	0.0336 (14)	0.0384 (15)	0.0484 (16)	−0.0036 (11)	0.0104 (12)	0.0030 (13)
N(12)	0.0333 (14)	0.0611 (19)	0.0413 (16)	0.0005 (13)	0.0135 (12)	−0.0041 (14)
C(1)	0.0435 (19)	0.0390 (18)	0.0415 (19)	−0.0008 (14)	0.0119 (15)	−0.0008 (15)
C(2)	0.053 (2)	0.0401 (18)	0.0392 (19)	−0.0049 (15)	0.0150 (16)	0.0008 (15)
C(3)	0.054 (2)	0.063 (2)	0.0341 (19)	−0.0035 (19)	−0.0005 (16)	0.0042 (18)
C(4)	0.0431 (18)	0.0308 (15)	0.0318 (16)	−0.0065 (13)	0.0082 (13)	0.0010 (13)
C(5)	0.0374 (17)	0.0411 (18)	0.0385 (18)	−0.0034 (14)	0.0045 (14)	0.0005 (15)
C(6)	0.0364 (17)	0.0347 (16)	0.0433 (18)	−0.0033 (13)	0.0074 (14)	−0.0026 (14)
C(7)	0.047 (2)	0.063 (2)	0.0380 (19)	0.0073 (18)	0.0116 (16)	0.0002 (17)
C(8)	0.048 (2)	0.066 (2)	0.048 (2)	0.0044 (19)	0.0182 (17)	−0.0013 (19)
C(9)	0.042 (2)	0.085 (3)	0.063 (2)	0.019 (2)	0.0084 (19)	0.000 (2)
C(10)	0.053 (2)	0.0403 (19)	0.046 (2)	−0.0089 (16)	−0.0012 (17)	0.0015 (16)
C(11)	0.044 (2)	0.0393 (19)	0.058 (2)	−0.0037 (16)	−0.0056 (17)	0.0087 (18)
C(12)	0.052 (2)	0.056 (2)	0.100 (3)	0.0067 (19)	0.035 (2)	0.013 (2)
C(13)	0.0368 (17)	0.0288 (15)	0.0432 (18)	−0.0054 (12)	0.0091 (14)	0.0008 (14)
C(14)	0.0447 (19)	0.0423 (19)	0.0395 (18)	−0.0092 (15)	0.0109 (15)	−0.0065 (15)
C(15)	0.0422 (18)	0.0312 (16)	0.0391 (18)	−0.0103 (13)	0.0027 (14)	−0.0001 (14)
C(16)	0.0362 (19)	0.0389 (19)	0.072 (2)	−0.0077 (15)	−0.0022 (18)	0.0017 (19)
C(17)	0.049 (2)	0.043 (2)	0.056 (2)	−0.0084 (17)	−0.0129 (18)	0.0064 (18)
C(18)	0.081 (3)	0.055 (2)	0.040 (2)	−0.018 (2)	0.006 (2)	−0.0049 (18)
C(19)	0.0357 (17)	0.0369 (17)	0.0399 (17)	−0.0049 (13)	0.0130 (14)	−0.0053 (14)
C(20)	0.0369 (17)	0.0372 (18)	0.048 (2)	−0.0017 (14)	0.0150 (15)	−0.0017 (15)
C(21)	0.049 (2)	0.057 (2)	0.042 (2)	−0.0015 (18)	0.0023 (16)	0.0024 (18)
C(22)	0.0351 (16)	0.0311 (15)	0.0331 (16)	−0.0044 (12)	0.0078 (13)	−0.0045 (13)
C(23)	0.0411 (18)	0.0405 (18)	0.0440 (19)	−0.0058 (14)	0.0159 (15)	−0.0092 (15)
C(24)	0.0314 (16)	0.0441 (19)	0.0356 (17)	0.0008 (13)	0.0083 (13)	−0.0019 (14)
C(25)	0.0355 (18)	0.054 (2)	0.064 (2)	−0.0065 (16)	0.0199 (17)	−0.0021 (19)
C(26)	0.042 (2)	0.080 (3)	0.061 (2)	−0.007 (2)	0.0266 (19)	0.000 (2)
C(27)	0.047 (2)	0.089 (3)	0.051 (2)	−0.002 (2)	0.0214 (18)	−0.022 (2)
C(28)	0.059 (2)	0.068 (2)	0.048 (2)	−0.003 (2)	0.0191 (19)	0.001 (2)
C(29)	0.048 (2)	0.071 (2)	0.066 (2)	0.001 (2)	0.018 (2)	0.009 (2)

### Geometric parameters (Å, °)

**Table:**

Cu(1)—N(1)	2.042 (2)	N(12)—C(27)	1.459 (5)
Cu(1)—N(3)	1.958 (3)	C(1)—C(2)	1.352 (4)
Cu(1)—N(9)	1.914 (2)	C(4)—C(5)	1.488 (5)
Cu(2)—N(5)	2.049 (3)	C(5)—C(6)	1.491 (5)
Cu(2)—N(7)	1.935 (3)	C(7)—C(8)	1.344 (5)
Cu(2)—N(11)	1.916 (3)	C(10)—C(11)	1.338 (6)
S(1)—O(1)	1.423 (3)	C(13)—C(14)	1.492 (4)
S(1)—O(2)	1.416 (3)	C(14)—C(15)	1.495 (4)
S(1)—O(3)	1.430 (2)	C(16)—C(17)	1.341 (6)
S(1)—C(28)	1.811 (4)	C(19)—C(20)	1.356 (4)
S(2)—O(4)	1.427 (2)	C(22)—C(23)	1.486 (5)
S(2)—O(5)	1.422 (3)	C(23)—C(24)	1.496 (5)
S(2)—O(6)	1.430 (3)	C(25)—C(26)	1.349 (6)
S(2)—C(29)	1.807 (5)	C(1)—H(1)	0.950
F(1)—C(28)	1.331 (4)	C(2)—H(2)	0.950
F(2)—C(28)	1.307 (5)	C(3)—H(3A)	0.980
F(3)—C(28)	1.310 (6)	C(3)—H(3B)	0.980
F(4)—C(29)	1.312 (5)	C(3)—H(3C)	0.980
F(5)—C(29)	1.329 (4)	C(5)—H(5A)	0.990
F(6)—C(29)	1.337 (6)	C(5)—H(5B)	0.990
N(1)—C(1)	1.383 (5)	C(7)—H(7)	0.950
N(1)—C(4)	1.326 (3)	C(8)—H(8)	0.950
N(2)—C(2)	1.378 (5)	C(9)—H(9A)	0.980
N(2)—C(3)	1.462 (4)	C(9)—H(9B)	0.980
N(2)—C(4)	1.351 (4)	C(9)—H(9C)	0.980
N(3)—C(6)	1.322 (3)	C(10)—H(10)	0.950
N(3)—C(7)	1.388 (5)	C(11)—H(11)	0.950
N(4)—C(6)	1.346 (5)	C(12)—H(12A)	0.980
N(4)—C(8)	1.373 (5)	C(12)—H(12B)	0.980
N(4)—C(9)	1.459 (4)	C(12)—H(12C)	0.980
N(5)—C(10)	1.390 (4)	C(14)—H(14A)	0.990
N(5)—C(13)	1.314 (5)	C(14)—H(14B)	0.990
N(6)—C(11)	1.377 (5)	C(16)—H(16)	0.950
N(6)—C(12)	1.458 (6)	C(17)—H(17)	0.950
N(6)—C(13)	1.364 (4)	C(18)—H(18A)	0.980
N(7)—C(15)	1.330 (5)	C(18)—H(18B)	0.980
N(7)—C(16)	1.392 (4)	C(18)—H(18C)	0.980
N(8)—C(15)	1.359 (4)	C(19)—H(19)	0.950
N(8)—C(17)	1.368 (5)	C(20)—H(20)	0.950
N(8)—C(18)	1.461 (6)	C(21)—H(21A)	0.980
N(9)—C(19)	1.385 (4)	C(21)—H(21B)	0.980
N(9)—C(22)	1.333 (4)	C(21)—H(21C)	0.980
N(10)—C(20)	1.374 (5)	C(23)—H(23A)	0.990
N(10)—C(21)	1.462 (4)	C(23)—H(23B)	0.990
N(10)—C(22)	1.345 (4)	C(25)—H(25)	0.950
N(11)—C(24)	1.327 (4)	C(26)—H(26)	0.950
N(11)—C(25)	1.382 (5)	C(27)—H(27A)	0.980
N(12)—C(24)	1.354 (5)	C(27)—H(27B)	0.980
N(12)—C(26)	1.367 (5)	C(27)—H(27C)	0.980
			
N(1)—Cu(1)—N(3)	95.56 (12)	S(1)—C(28)—F(1)	110.9 (3)
N(1)—Cu(1)—N(9)	121.37 (12)	S(1)—C(28)—F(2)	112.6 (3)
N(3)—Cu(1)—N(9)	142.95 (12)	S(1)—C(28)—F(3)	112.6 (3)
N(5)—Cu(2)—N(7)	93.66 (12)	F(1)—C(28)—F(2)	106.2 (3)
N(5)—Cu(2)—N(11)	115.27 (11)	F(1)—C(28)—F(3)	105.7 (3)
N(7)—Cu(2)—N(11)	150.78 (12)	F(2)—C(28)—F(3)	108.3 (4)
O(1)—S(1)—O(2)	115.4 (2)	S(2)—C(29)—F(4)	112.4 (3)
O(1)—S(1)—O(3)	114.9 (2)	S(2)—C(29)—F(5)	111.7 (3)
O(1)—S(1)—C(28)	103.5 (2)	S(2)—C(29)—F(6)	110.8 (3)
O(2)—S(1)—O(3)	113.9 (2)	F(4)—C(29)—F(5)	107.1 (3)
O(2)—S(1)—C(28)	104.2 (2)	F(4)—C(29)—F(6)	107.1 (3)
O(3)—S(1)—C(28)	102.8 (2)	F(5)—C(29)—F(6)	107.4 (4)
O(4)—S(2)—O(5)	114.6 (2)	N(1)—C(1)—H(1)	124.9
O(4)—S(2)—O(6)	115.6 (2)	C(2)—C(1)—H(1)	124.9
O(4)—S(2)—C(29)	102.7 (2)	N(2)—C(2)—H(2)	127.2
O(5)—S(2)—O(6)	115.13 (19)	C(1)—C(2)—H(2)	127.2
O(5)—S(2)—C(29)	102.3 (2)	N(2)—C(3)—H(3A)	109.5
O(6)—S(2)—C(29)	103.9 (2)	N(2)—C(3)—H(3B)	109.5
Cu(1)—N(1)—C(1)	130.7 (2)	N(2)—C(3)—H(3C)	109.5
Cu(1)—N(1)—C(4)	121.9 (2)	H(3A)—C(3)—H(3B)	109.5
C(1)—N(1)—C(4)	105.7 (2)	H(3A)—C(3)—H(3C)	109.5
C(2)—N(2)—C(3)	125.3 (3)	H(3B)—C(3)—H(3C)	109.5
C(2)—N(2)—C(4)	107.9 (2)	C(4)—C(5)—H(5A)	107.6
C(3)—N(2)—C(4)	126.7 (3)	C(4)—C(5)—H(5B)	107.6
Cu(1)—N(3)—C(6)	124.5 (2)	C(6)—C(5)—H(5A)	107.6
Cu(1)—N(3)—C(7)	129.8 (2)	C(6)—C(5)—H(5B)	107.6
C(6)—N(3)—C(7)	105.5 (3)	H(5A)—C(5)—H(5B)	107.1
C(6)—N(4)—C(8)	107.6 (2)	N(3)—C(7)—H(7)	125.1
C(6)—N(4)—C(9)	126.5 (3)	C(8)—C(7)—H(7)	125.1
C(8)—N(4)—C(9)	125.9 (3)	N(4)—C(8)—H(8)	126.9
Cu(2)—N(5)—C(10)	129.6 (2)	C(7)—C(8)—H(8)	126.8
Cu(2)—N(5)—C(13)	122.3 (2)	N(4)—C(9)—H(9A)	109.5
C(10)—N(5)—C(13)	106.2 (3)	N(4)—C(9)—H(9B)	109.5
C(11)—N(6)—C(12)	126.1 (3)	N(4)—C(9)—H(9C)	109.5
C(11)—N(6)—C(13)	107.1 (3)	H(9A)—C(9)—H(9B)	109.5
C(12)—N(6)—C(13)	126.7 (3)	H(9A)—C(9)—H(9C)	109.5
Cu(2)—N(7)—C(15)	124.2 (2)	H(9B)—C(9)—H(9C)	109.5
Cu(2)—N(7)—C(16)	129.7 (3)	N(5)—C(10)—H(10)	125.2
C(15)—N(7)—C(16)	105.7 (3)	C(11)—C(10)—H(10)	125.2
C(15)—N(8)—C(17)	107.6 (3)	N(6)—C(11)—H(11)	126.7
C(15)—N(8)—C(18)	126.1 (3)	C(10)—C(11)—H(11)	126.7
C(17)—N(8)—C(18)	126.1 (3)	N(6)—C(12)—H(12A)	109.5
Cu(1)—N(9)—C(19)	128.0 (2)	N(6)—C(12)—H(12B)	109.5
Cu(1)—N(9)—C(22)	126.2 (2)	N(6)—C(12)—H(12C)	109.5
C(19)—N(9)—C(22)	105.7 (2)	H(12A)—C(12)—H(12B)	109.5
C(20)—N(10)—C(21)	124.5 (2)	H(12A)—C(12)—H(12C)	109.5
C(20)—N(10)—C(22)	107.7 (2)	H(12B)—C(12)—H(12C)	109.5
C(21)—N(10)—C(22)	127.7 (3)	C(13)—C(14)—H(14A)	108.1
Cu(2)—N(11)—C(24)	131.4 (2)	C(13)—C(14)—H(14B)	108.1
Cu(2)—N(11)—C(25)	123.0 (2)	C(15)—C(14)—H(14A)	108.1
C(24)—N(11)—C(25)	105.5 (3)	C(15)—C(14)—H(14B)	108.2
C(24)—N(12)—C(26)	107.3 (3)	H(14A)—C(14)—H(14B)	107.3
C(24)—N(12)—C(27)	125.6 (3)	N(7)—C(16)—H(16)	125.2
C(26)—N(12)—C(27)	126.9 (3)	C(17)—C(16)—H(16)	125.2
N(1)—C(1)—C(2)	110.2 (3)	N(8)—C(17)—H(17)	126.5
N(2)—C(2)—C(1)	105.7 (3)	C(16)—C(17)—H(17)	126.5
N(1)—C(4)—N(2)	110.6 (3)	N(8)—C(18)—H(18A)	109.5
N(1)—C(4)—C(5)	129.1 (3)	N(8)—C(18)—H(18B)	109.5
N(2)—C(4)—C(5)	120.3 (2)	N(8)—C(18)—H(18C)	109.5
C(4)—C(5)—C(6)	118.8 (2)	H(18A)—C(18)—H(18B)	109.5
N(3)—C(6)—N(4)	110.9 (3)	H(18A)—C(18)—H(18C)	109.5
N(3)—C(6)—C(5)	129.1 (3)	H(18B)—C(18)—H(18C)	109.5
N(4)—C(6)—C(5)	120.0 (2)	N(9)—C(19)—H(19)	125.3
N(3)—C(7)—C(8)	109.7 (3)	C(20)—C(19)—H(19)	125.3
N(4)—C(8)—C(7)	106.3 (3)	N(10)—C(20)—H(20)	126.8
N(5)—C(10)—C(11)	109.6 (3)	C(19)—C(20)—H(20)	126.8
N(6)—C(11)—C(10)	106.6 (3)	N(10)—C(21)—H(21A)	109.5
N(5)—C(13)—N(6)	110.4 (2)	N(10)—C(21)—H(21B)	109.5
N(5)—C(13)—C(14)	128.9 (3)	N(10)—C(21)—H(21C)	109.5
N(6)—C(13)—C(14)	120.6 (3)	H(21A)—C(21)—H(21B)	109.5
C(13)—C(14)—C(15)	116.6 (3)	H(21A)—C(21)—H(21C)	109.5
N(7)—C(15)—N(8)	110.2 (3)	H(21B)—C(21)—H(21C)	109.5
N(7)—C(15)—C(14)	130.1 (2)	C(22)—C(23)—H(23A)	108.4
N(8)—C(15)—C(14)	119.7 (3)	C(22)—C(23)—H(23B)	108.4
N(7)—C(16)—C(17)	109.5 (3)	C(24)—C(23)—H(23A)	108.4
N(8)—C(17)—C(16)	106.9 (3)	C(24)—C(23)—H(23B)	108.4
N(9)—C(19)—C(20)	109.4 (3)	H(23A)—C(23)—H(23B)	107.4
N(10)—C(20)—C(19)	106.4 (3)	N(11)—C(25)—H(25)	125.2
N(9)—C(22)—N(10)	110.8 (3)	C(26)—C(25)—H(25)	125.2
N(9)—C(22)—C(23)	124.1 (2)	N(12)—C(26)—H(26)	126.7
N(10)—C(22)—C(23)	125.0 (2)	C(25)—C(26)—H(26)	126.7
C(22)—C(23)—C(24)	115.6 (2)	N(12)—C(27)—H(27A)	109.5
N(11)—C(24)—N(12)	110.8 (3)	N(12)—C(27)—H(27B)	109.5
N(11)—C(24)—C(23)	128.2 (3)	N(12)—C(27)—H(27C)	109.5
N(12)—C(24)—C(23)	121.0 (3)	H(27A)—C(27)—H(27B)	109.5
N(11)—C(25)—C(26)	109.7 (3)	H(27A)—C(27)—H(27C)	109.5
N(12)—C(26)—C(25)	106.7 (4)	H(27B)—C(27)—H(27C)	109.5
			
N(1)—Cu(1)—N(3)—C(6)	−1.1 (2)	Cu(2)—N(5)—C(10)—C(11)	163.8 (2)
N(1)—Cu(1)—N(3)—C(7)	174.5 (3)	Cu(2)—N(5)—C(13)—N(6)	−165.2 (2)
N(3)—Cu(1)—N(1)—C(1)	171.7 (2)	Cu(2)—N(5)—C(13)—C(14)	14.8 (4)
N(3)—Cu(1)—N(1)—C(4)	9.0 (2)	C(10)—N(5)—C(13)—N(6)	0.4 (3)
N(1)—Cu(1)—N(9)—C(19)	5.9 (3)	C(10)—N(5)—C(13)—C(14)	−179.6 (3)
N(1)—Cu(1)—N(9)—C(22)	−177.2 (2)	C(13)—N(5)—C(10)—C(11)	−0.3 (4)
N(9)—Cu(1)—N(1)—C(1)	−11.5 (3)	C(12)—N(6)—C(11)—C(10)	−178.1 (3)
N(9)—Cu(1)—N(1)—C(4)	−174.2 (2)	C(11)—N(6)—C(13)—N(5)	−0.3 (3)
N(3)—Cu(1)—N(9)—C(19)	−179.4 (2)	C(11)—N(6)—C(13)—C(14)	179.7 (3)
N(3)—Cu(1)—N(9)—C(22)	−2.5 (3)	C(13)—N(6)—C(11)—C(10)	0.1 (3)
N(9)—Cu(1)—N(3)—C(6)	−176.5 (2)	C(12)—N(6)—C(13)—N(5)	177.9 (3)
N(9)—Cu(1)—N(3)—C(7)	−1.0 (4)	C(12)—N(6)—C(13)—C(14)	−2.2 (5)
N(5)—Cu(2)—N(7)—C(15)	17.9 (2)	Cu(2)—N(7)—C(15)—N(8)	173.6 (2)
N(5)—Cu(2)—N(7)—C(16)	−170.1 (3)	Cu(2)—N(7)—C(15)—C(14)	−7.2 (5)
N(7)—Cu(2)—N(5)—C(10)	176.6 (3)	Cu(2)—N(7)—C(16)—C(17)	−172.7 (2)
N(7)—Cu(2)—N(5)—C(13)	−21.6 (2)	C(15)—N(7)—C(16)—C(17)	0.4 (4)
N(5)—Cu(2)—N(11)—C(24)	−80.3 (3)	C(16)—N(7)—C(15)—N(8)	0.0 (3)
N(5)—Cu(2)—N(11)—C(25)	102.8 (2)	C(16)—N(7)—C(15)—C(14)	179.2 (3)
N(11)—Cu(2)—N(5)—C(10)	0.8 (3)	C(15)—N(8)—C(17)—C(16)	0.6 (4)
N(11)—Cu(2)—N(5)—C(13)	162.7 (2)	C(17)—N(8)—C(15)—N(7)	−0.4 (4)
N(7)—Cu(2)—N(11)—C(24)	108.3 (3)	C(17)—N(8)—C(15)—C(14)	−179.7 (3)
N(7)—Cu(2)—N(11)—C(25)	−68.6 (3)	C(18)—N(8)—C(15)—N(7)	−176.3 (3)
N(11)—Cu(2)—N(7)—C(15)	−169.9 (2)	C(18)—N(8)—C(15)—C(14)	4.4 (5)
N(11)—Cu(2)—N(7)—C(16)	2.1 (4)	C(18)—N(8)—C(17)—C(16)	176.5 (3)
O(1)—S(1)—C(28)—F(1)	62.2 (3)	Cu(1)—N(9)—C(19)—C(20)	178.1 (2)
O(1)—S(1)—C(28)—F(2)	−179.0 (3)	Cu(1)—N(9)—C(22)—N(10)	−177.9 (2)
O(1)—S(1)—C(28)—F(3)	−56.2 (3)	Cu(1)—N(9)—C(22)—C(23)	6.2 (4)
O(2)—S(1)—C(28)—F(1)	−58.9 (3)	C(19)—N(9)—C(22)—N(10)	−0.5 (3)
O(2)—S(1)—C(28)—F(2)	59.9 (3)	C(19)—N(9)—C(22)—C(23)	−176.4 (3)
O(2)—S(1)—C(28)—F(3)	−177.2 (2)	C(22)—N(9)—C(19)—C(20)	0.8 (3)
O(3)—S(1)—C(28)—F(1)	−178.0 (3)	C(21)—N(10)—C(20)—C(19)	178.7 (3)
O(3)—S(1)—C(28)—F(2)	−59.1 (4)	C(20)—N(10)—C(22)—N(9)	0.0 (3)
O(3)—S(1)—C(28)—F(3)	63.7 (3)	C(20)—N(10)—C(22)—C(23)	175.9 (3)
O(4)—S(2)—C(29)—F(4)	−55.1 (3)	C(22)—N(10)—C(20)—C(19)	0.5 (3)
O(4)—S(2)—C(29)—F(5)	−175.5 (3)	C(21)—N(10)—C(22)—N(9)	−178.1 (3)
O(4)—S(2)—C(29)—F(6)	64.7 (3)	C(21)—N(10)—C(22)—C(23)	−2.2 (5)
O(5)—S(2)—C(29)—F(4)	64.0 (3)	Cu(2)—N(11)—C(24)—N(12)	−177.3 (2)
O(5)—S(2)—C(29)—F(5)	−56.5 (4)	Cu(2)—N(11)—C(24)—C(23)	−1.0 (5)
O(5)—S(2)—C(29)—F(6)	−176.3 (2)	Cu(2)—N(11)—C(25)—C(26)	176.7 (2)
O(6)—S(2)—C(29)—F(4)	−175.9 (3)	C(24)—N(11)—C(25)—C(26)	−0.9 (3)
O(6)—S(2)—C(29)—F(5)	63.7 (4)	C(25)—N(11)—C(24)—N(12)	−0.0 (3)
O(6)—S(2)—C(29)—F(6)	−56.1 (3)	C(25)—N(11)—C(24)—C(23)	176.2 (3)
Cu(1)—N(1)—C(1)—C(2)	−165.4 (2)	C(24)—N(12)—C(26)—C(25)	−1.4 (4)
Cu(1)—N(1)—C(4)—N(2)	167.0 (2)	C(26)—N(12)—C(24)—N(11)	0.9 (3)
Cu(1)—N(1)—C(4)—C(5)	−11.9 (4)	C(26)—N(12)—C(24)—C(23)	−175.7 (2)
C(1)—N(1)—C(4)—N(2)	0.6 (3)	C(27)—N(12)—C(24)—N(11)	177.4 (3)
C(1)—N(1)—C(4)—C(5)	−178.4 (3)	C(27)—N(12)—C(24)—C(23)	0.9 (4)
C(4)—N(1)—C(1)—C(2)	−0.5 (3)	C(27)—N(12)—C(26)—C(25)	−177.9 (3)
C(3)—N(2)—C(2)—C(1)	177.6 (3)	N(1)—C(1)—C(2)—N(2)	0.3 (4)
C(2)—N(2)—C(4)—N(1)	−0.4 (3)	N(1)—C(4)—C(5)—C(6)	4.6 (5)
C(2)—N(2)—C(4)—C(5)	178.7 (3)	N(2)—C(4)—C(5)—C(6)	−174.2 (3)
C(4)—N(2)—C(2)—C(1)	0.0 (3)	C(4)—C(5)—C(6)—N(3)	5.5 (5)
C(3)—N(2)—C(4)—N(1)	−178.0 (3)	C(4)—C(5)—C(6)—N(4)	−177.0 (3)
C(3)—N(2)—C(4)—C(5)	1.1 (5)	N(3)—C(7)—C(8)—N(4)	−0.2 (3)
Cu(1)—N(3)—C(6)—N(4)	176.7 (2)	N(5)—C(10)—C(11)—N(6)	0.1 (3)
Cu(1)—N(3)—C(6)—C(5)	−5.6 (5)	N(5)—C(13)—C(14)—C(15)	2.1 (5)
Cu(1)—N(3)—C(7)—C(8)	−176.2 (2)	N(6)—C(13)—C(14)—C(15)	−177.9 (2)
C(6)—N(3)—C(7)—C(8)	−0.0 (3)	C(13)—C(14)—C(15)—N(7)	−7.0 (5)
C(7)—N(3)—C(6)—N(4)	0.3 (4)	C(13)—C(14)—C(15)—N(8)	172.1 (3)
C(7)—N(3)—C(6)—C(5)	178.0 (3)	N(7)—C(16)—C(17)—N(8)	−0.6 (4)
C(6)—N(4)—C(8)—C(7)	0.4 (4)	N(9)—C(19)—C(20)—N(10)	−0.8 (3)
C(8)—N(4)—C(6)—N(3)	−0.4 (4)	N(9)—C(22)—C(23)—C(24)	−84.8 (3)
C(8)—N(4)—C(6)—C(5)	−178.3 (3)	N(10)—C(22)—C(23)—C(24)	99.9 (3)
C(9)—N(4)—C(6)—N(3)	179.7 (3)	C(22)—C(23)—C(24)—N(11)	23.6 (4)
C(9)—N(4)—C(6)—C(5)	1.8 (5)	C(22)—C(23)—C(24)—N(12)	−160.4 (2)
C(9)—N(4)—C(8)—C(7)	−179.7 (3)	N(11)—C(25)—C(26)—N(12)	1.4 (4)
